# Autoimmunity, cancer and COVID-19 abnormally activate wound healing pathways: critical role of inflammation

**DOI:** 10.1007/s00418-022-02140-x

**Published:** 2022-07-22

**Authors:** Peter Gál, Jan Brábek, Michal Holub, Milan Jakubek, Aleksi Šedo, Lukáš Lacina, Karolína Strnadová, Petr Dubový, Helena Hornychová, Aleš Ryška, Karel Smetana

**Affiliations:** 1 Department of Pharmacology, Pavol Jozef Šafárik University, Košice, Slovak Republic; 2 Department of Biomedical Research, East-Slovak Institute of Cardiovascular Diseases, Košice, Slovak Republic; 3 Prague Burn Centre, Third Faculty of Medicine, Charles University and University Hospital Kralovske Vinohrady, Prague, Czech Republic; 4 Department of Cell Biology, Faculty of Science, Charles University, 120 00 Prague 2, Czech Republic; 5 BIOCEV, Faculty of Science, Charles University, 252 50 Vestec, Czech Republic; 6 Department of Infectious Diseases, First Faculty of Medicine, Military University Hospital Prague and Charles University, 160 00 Prague, Czech Republic; 7 Department of Paediatrics and Adolescent Medicine, First Faculty of Medicine, Charles University, 120 00 Prague 2, Czech Republic; 8 BIOCEV, First Faculty of Medicine, Charles University, 252 50 Vestec, Czech Republic; 9 Department of Analytical Chemistry, University of Chemistry and Technology Prague, 166 28 Prague 6, Czech Republic; 10 Institute of Biochemistry and Experimental Oncology, First Faculty of Medicine, Charles University, 120 00 Praha 2, Czech Republic; 11 Institute of Anatomy, First Faculty of Medicine, Charles University, 120 00 Prague 2, Czech Republic; 12 Department of Dermatovenereology, First Faculty of Medicine, Charles University, 120 00 Prague 2, Czech Republic; 13 Institute of Anatomy, Faculty of Medicine, Masaryk University, 625 00 Brno, Czech Republic; 14 The Fingerland Department of Pathology, Faculty of Medicine Hradec Králové, Charles University, 500 05 Hradec Králové, Czech Republic

**Keywords:** Wound healing, Granulation tissue, Peripheral nerve injury, Rheumatoid arthritis, Cancer stroma, SARS-CoV-2, Myofibroblast, IL-6, Inflammation

## Abstract

Recent evidence indicates that targeting IL-6 provides broad therapeutic approaches to several diseases. In patients with cancer, autoimmune diseases, severe respiratory infections [e.g. coronavirus disease 2019 (COVID-19)] and wound healing, IL-6 plays a critical role in modulating the systemic and local microenvironment. Elevated serum levels of IL-6 interfere with the systemic immune response and are associated with disease progression and prognosis. As already noted, monoclonal antibodies blocking either IL-6 or binding of IL-6 to receptors have been used/tested successfully in the treatment of rheumatoid arthritis, many cancer types, and COVID-19. Therefore, in the present review, we compare the impact of IL-6 and anti-IL-6 therapy to demonstrate common (pathological) features of the studied diseases such as formation of granulation tissue with the presence of myofibroblasts and deposition of new extracellular matrix. We also discuss abnormal activation of other wound-healing-related pathways that have been implicated in autoimmune disorders, cancer or COVID-19.

## Introduction

“Tumours are wounds that do not heal” (Dvorak 1986). Based on gross morphological resemblance, this classical parallel was proposed by Harold F. Dvorak more than three decades ago (in 1986). Notably, the tumour stroma of the vast majority of solid cancers is very similar to the granulation tissue that is indispensable for physiological wound healing. Differences are sometimes very subtle and given by the context. Indeed, since recently, we can follow a multitude of identical molecular mechanisms underlying these pathological conditions and thus supporting this historical parallel more robustly. Therefore, a broader view seems advantageous in distinguishing what makes one mechanism beneficial in healing, while the identical mechanism may represent an advantage for cancer progression and a critical obstacle in the treatment of malignancies.

The COVID-19 pandemic in recent years brought many new challenging inputs to the clinics and biomedical sciences, including pathology. Respiratory complications in severe acute respiratory syndrome coronavirus 2 (SARS-CoV-2) infection are associated with substantial morbidity and mortality. Interestingly, histopathological examination of pulmonary injury in patients who died from COVID-19 revealed striking morphological similarities to findings in autoimmune diseases, cancer and wound healing (Giacomelli et al. 2021). Hence, in the present review, we define the overlapping histological reaction patterns observed in this clinically heterogeneous and previously not compared group of diseases, emphasizing interleukin (IL)-6 signalling.

From a functional point of view, autoimmunity, cancer, COVID-19 and wound healing share several common features represented by deregulated inflammatory/immune responses. Inflammatory cytokine profiles of patients suffering from severe and critical forms of COVID-19 or various types of cancer revealed a potential crossing point, namely significantly elevated serum levels of pro-inflammatory cytokine IL-6 (Brábek et al. 2020). Accordingly, we proposed IL-6 inhibition as a potential therapeutic strategy for COVID-19-related cytokine release syndrome (Smetana and Brábek 2020; Smetana et al. 2020b). This idea was supported by pre-existing permission to use the inhibitor in human medicine [approved by the US Food and Drug Administration (FDA) and European Medicines Agency (EMA) for the treatment of other conditions]. Indeed, this approach was later adopted to treat severe forms of COVID-19 (Lamontagne et al. 2020). However, clinical results achieved with IL-6 signalling inhibitors are somewhat conflicting in critically ill patients with COVID-19 (Declercq et al. 2021; Majidpoor and Mortezaee 2022). At this point, we can hypothesize that a more stringent definition of inclusion/exclusion criteria of patient enrolment would be beneficial to evaluate the outcome of therapy more objectively. On the other hand, optimal drug timing during combination therapies, which is still not well known and seems to be somewhat underrated, may improve the therapeutic outcomes. In this context, it seems likely that anti-IL-6 therapy can offer certain benefits in at least some patients with COVID-19 if properly indicated.

## Skin injury – an example of a simple healing process

Undamaged skin represents a protective barrier against a potentially harmful external environment. The skin barrier restricts the invasion of pathogenic microorganisms (bacteria, fungi, viruses) and prevents the loss of water, crystalloids and proteins. Therefore, acute and chronic wounds pose serious healthcare issues.

Skin injury is an excellent model to study the general aspects of wound healing. When the skin integrity barrier is broken, the barrier function is compromised. The healing process requires an effective interplay of various cells regulated by the timely production of molecular signals to replace the missing tissue. More specifically, the skin is a valuable model for studying the complex epithelial–mesenchymal interactions between the epidermis and dermis. The precisely regulated interplay between regeneration of the epidermis and fibroplastic response of the dermis is a prerequisite for successful healing. Deeper insights into the aspects of healing mechanisms may facilitate the identification of bioinspired therapeutic options (Pollini and Paladini 2020).

Of note, the interfollicular epidermis can regenerate fully in humans. However, dermal architecture repair is not so easy in humans, which leads to scar production. Dermis repair is divided into four predictable phases: blood clotting/haemostasis (which may be part of inflammation), inflammation, proliferation and maturation/remodelling. Once this sequence is completed, re-epithelization finally reconstitutes the barrier integrity (Pastar et al. 2014). Although wound healing is complex and tightly orchestrated, it is also susceptible to interruption and failure, resulting in non-healing/chronic wounds or pathological scars (Čoma et al. 2021).

Traumatic tissue injury is usually associated with haemorrhage resulting in the formation of a stable haemostatic clot of polymerized fibrin, preventing major blood loss by a process called haemostasis. The coagulum temporarily fills the defect (forming a crust) and provisionally protects deeper tissue. The formation of this temporary barrier is initiated by the intrinsic (platelets) and extrinsic coagulation (clotting factors) cascade (Reinke and Sorg 2012).

Following haemostasis, dilatation and permeabilization of vessels allow leucocytes to reach the injury site; thus, the inflammatory phase begins. The main goal of the invading leucocytes is to eliminate pathogens, resolve the inflammation and remove necrotic cells/tissue (Ellis et al. 2018). Immune cells are responsible for removing the tissue debris and preventing wound colonization by microorganisms. These immune cells mainly include granulocytes (neutrophils and eosinophils), macrophages, mast cells, natural killer (NK) cells, B lymphocytes and subsets of T lymphocytes.

Neutrophils, the first and most abundant immune cells infiltrating the wound, play an essential role in debris and pathogen removal (de Oliveira et al. 2016). Monocytes/macrophages are of high importance in wound healing as they play a critical role in resolving acute inflammation. Depending on their polarization, macrophages can enhance the immune response (M1 polarization) or attenuate inflammation (M2 polarization) and coordinate tissue repair. The functional and morphologic outcome of wound healing is highly dependent on the success of inflammation resolution (Wilgus 2020). Persisting inflammation may lead to dysregulation of keratinocyte and fibroblast differentiation and often results in excessive scarring and, in the worst case, even in the formation of a hypertrophic/keloid scar (Landén et al. 2016).

To interact across the wound site and orchestrate the inflammatory activity, immune cells release a broad panel of bioactive factors. These include, but are not limited to, transforming growth factor (TGF)-β, tumour necrosis factor (TNF)-α, interferon (IFN)-γ, vascular endothelial growth factor A (VEGFA), basic fibroblast growth factor (bFGF), a panel of interleukins (IL-4-6, IL-9-13, IL-17, IL-23), chemokines [chemokine C–X–C ligand motif (CXCL)-8, CXCL-12] and numerous proteases (Čoma et al. 2021).

Of note, some of these factors are not produced in the microenvironment specifically by a single cell type. To exemplify this pleiotropy, IL-6 is produced by neutrophils, macrophages, mast cells and T lymphocytes. Similarly, immune cells also widely produce members of the TGF-β family. These factors influence different immune cells in the wound microenvironment simultaneously. Moreover, these signals inevitably also affect local fibroblasts, epithelial cells, and endothelium of capillaries.

Moreover, these cytokines can also leak into the systemic circulation. Measurements of IL-6 serum levels were used to monitor large wound healing (Avazi et al. 2019; Zhang et al. 2020a). Hyperactivation of the immune system was rarely observed in large, infected wounds or extensive burn injury, leading anecdotally to the so-called cytokine storm syndrome (Adamik et al. 1997; Mulder et al. 2021).

Successful resolution of the inflammation enables healing to enter the proliferation phase approximately 2–4 days post-injury (Wokalek and Ruh 1991). The phase includes formation of the so-called granulation tissue. This provisional tissue is composed of persisting recruited inflammatory/immune cells, sprouting endothelium of capillaries and fibroblasts producing extracellular matrix (ECM). Fibroblasts first produce a loose ECM that provides a temporary provisional scaffold for other migrating and proliferating cells (Tracy et al. 2016).

Surprisingly, dermal fibroblasts are a highly heterogeneous population of mesenchymal cells. From a histological point of view, two principal subgroups of fibroblasts, papillary and reticular, have been identified. These two pools differ in their proliferative capacity, ability to contract wounds, and expression profile. Single-cell sequencing further distinguished up to six functionally different subsets of dermal fibroblasts. However, only the population expressing dipeptidyl-peptidase IV (also known as CD26) was responsible for ECM production, and thus could play a crucial role in new tissue formation during wound healing (Vorstandlechner et al. 2020). As expected, the resulting scar is highly dependent on the fibroblast subset present in the wounded site (Griffin et al. 2020).

Of note, such remarkable fibroblast diversity can also result from the developmental origin (Thulabandu et al. 2018). In the craniofacial region, dermal fibroblasts have potentially a dual embryonic origin of cranial neural crest (facial fibroblasts) and cephalic mesoderm. Fibroblasts present in the dorsal skin originate from the somites. Finally, fibroblasts in the ventral flank and limb dermis originate from the lateral plate mesoderm (Wong et al. 2006).

Intriguingly, fibroblasts or fibroblast-like cells can also arise from various cell populations. This includes origin from epithelial cells by epithelial-to-mesenchymal transition (EMT) (Thiery et al. 2009), epidermal stem cells (Li et al. 2016), bone marrow-derived mesenchymal stem cells (Saikia et al. 2020) and circulating fibrocytes (Quan et al. 2006) as well as from endothelial cells by endothelial-to-mesenchymal transition (EndMT) (Piera-Velazquez et al. 2011).

**Fig. 1:**
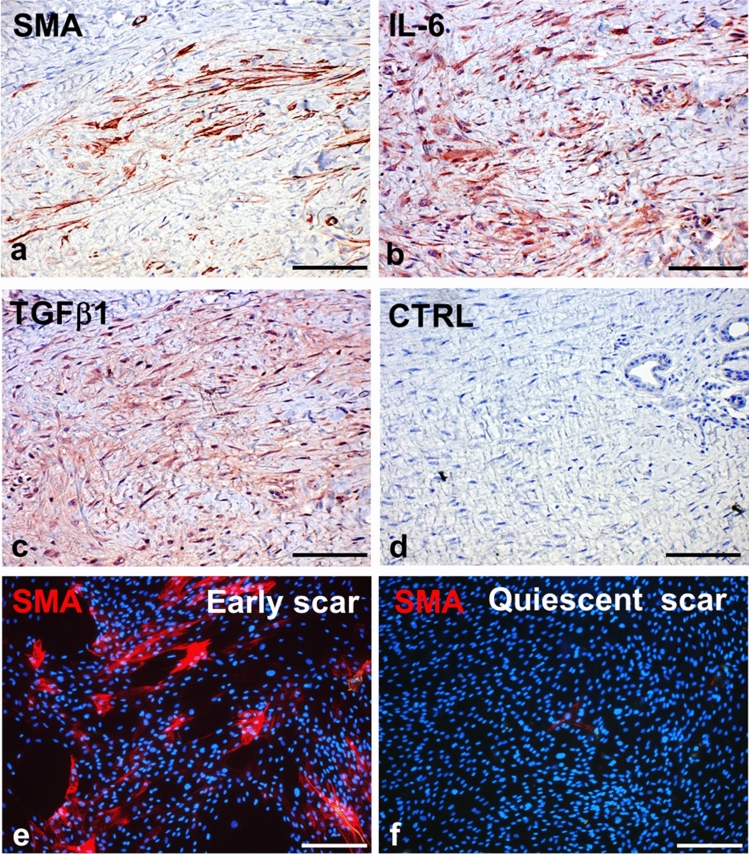
Active scar contains numerous α-smooth muscle actin (SMA)-positive myofibroblasts surrounded by extracellular matrix (**a**). Myofibroblasts and infiltrating immune cells produce IL-6 (**b**) and TGF-β1 (**c**); specificity of the reaction was verified by negative (isotype) control (**d**). While numerous SMA-positive fibroblasts can be isolated from an early active scar (**e**), the fibroblasts prepared from a quiescent scar are devoid of SMA expression (**f**). Nuclei are counterstained with DAPI (scale bar, 100 µm)

For the wound contraction, it is important that fibroblasts can differentiate into myofibroblasts (Hinz 2016) containing stress fibres allowing them to generate contractile forces facilitating wound closure (Li and Wang 2011; Chitturi et al. 2015). Several cytokines, growth factors, and growth and adhesion-regulating galectins were identified as fibroblast and myofibroblast stimulators. These include TGF-β1, CTGF, FGF, PDGF, IGF and galectin-1 (Bonner et al. 1990; Clark et al. 1997; Grazul-Bilska et al. 2002; Dvořánková et al. 2011; Hung et al. 2013; Lin et al. 2015). Of the cytokines listed above, TGF-β1 has been repeatedly reported as a critical factor for fibroblast differentiation into myofibroblasts (Evans et al. 2003; Zhang et al. 2020b; Macarak et al. 2021) (Fig. 1).

In parallel, a process called angiogenesis is in progress. This leads to the formation of new blood vessels, providing nutrients and oxygen to satisfy the demand of rapidly proliferating cells (Eelen et al. 2020). VEGF is the most studied and well-known pro-angiogenic molecule. VEGF was identified as vascular endothelial cell mitogen and regulator of endothelial integrin expression during vessel sprouting. Notably, VEGF may act as a chemokine for macrophages, directly activating them via the VEGF receptor (VEGFR) (Barleon et al. 1996). Therefore, VEGF may be considered as an indirect pro-inflammatory cytokine that promotes excessive scar formation.

The cell interactions occurring during the proliferation phase, namely epithelial–mesenchymal cross-talk between fibroblasts, endothelial cells and keratinocytes, have a major impact on the consequent maturation/remodelling phase of wound healing. Any failure of these interactions can lead to a sub-optimal healing result. The resulting scar may be clinically almost unnoticeable in neonates; however, it is usually more apparent in adults (Borsky et al. 2012). Although this unequal healing capacity observed across different ages is a clinically well-documented phenomenon, its explanation is not simple.

The formation of granulation tissue gradually stops through cell apoptosis and converts the wound to avascular and acellular scar. Untimely, cessation of this process leads to excessive accumulation of fibrotic tissue and formation of, e.g., hypertrophic/keloid scarring. However, keloid formation also requires certain genetic susceptibility (Nyika et al. 2022).

Therefore, optimal healing is based on a dynamic equilibrium of ECM production and degradation by proteolytic enzymes (Čoma et al. 2021). It was proposed that the neonatal fibroblasts maintain some properties similar to mesenchymal stem cells (Mateu et al. 2016), also accompanied by deregulation of TGF-β signalling with low expression of the TGF-β II receptor (Živicová et al. 2017).

## Peripheral nerve injury – an example of wound healing in highly specialized tissue

As introduced above, human skin has a relatively simple structure and rapid turnover. It differs from the highly specialized structure of nerves with slow turnover. It is well known that the nervous system lacks some extensive capacity for neural regeneration in humans. Therefore, even peripheral nerve repair and regeneration remain among the greatest challenges in regenerative medicine. However, the staging of skin wound healing can be efficiently applied to the repair and/or regeneration of other body tissues, including peripheral nerves. Indeed, the mechanisms are conserved and surprisingly similar.

For example, the proximal and distal stumps of disconnected peripheral nerve exhibit structural changes analogical to the proliferation phase observed during skin wound healing. They are associated with granular disintegration of axons, including their myelin sheaths distal to the injury. Further, there is an accumulation of fibroblasts extensively producing collagen and appearance of myofibroblasts in the proximal stump. Neural scar formation is a part of the injured nerve regeneration, but the excessive expansion of intra-neural scar tissue often hampers nerve regeneration (Wang et al. 2019; Fertala et al. 2020). Of note, it was recently demonstrated that myofibroblasts play a critical role in the nerve outgrowth/regeneration into the gap of an end-to-end nerve suture (Katenkamp and Stiller 1978; Fertala et al. 2020). On the other hand, numerous myofibroblasts occur in the posttraumatic neuromas of the proximal stump of permanently interrupted nerves, induced by chronic inflammation (Oliveira et al. 2018), and there is a clinically evidenced association with extreme pain due to direct malignant infiltration of nerves (Yan et al. 2012) observed in patients with cancer (Urch and Dickenson 2008; Yeh et al. 2016).

A rapidly growing body of evidence suggests that the immune system and its products play a critical role in the regeneration of the peripheral nervous system (Lindborg et al. 2018; Zigmond and Echevarria 2019; Dubový et al. 2021). The role of immune cell recruitment was well exemplified by the involvement of macrophages in the regeneration of neurons (Niemi et al. 2016) and peripheral nerves (Wofford et al. 2022). In detail, the presence of pro-inflammatory cytokines during the first phase of Wallerian degeneration promotes recruitment of macrophages. Later, these macrophages turn into the M2 phenotype, resolving the inflammation via production of anti-inflammatory cytokines. Therefore, macrophage infiltration into an injured nerve is an essential step favouring regeneration. Indeed, transplantation of M2 macrophages attenuated neuropathy-induced mechanical hypersensitivity (Pannell et al. 2016).

**Fig. 2:**
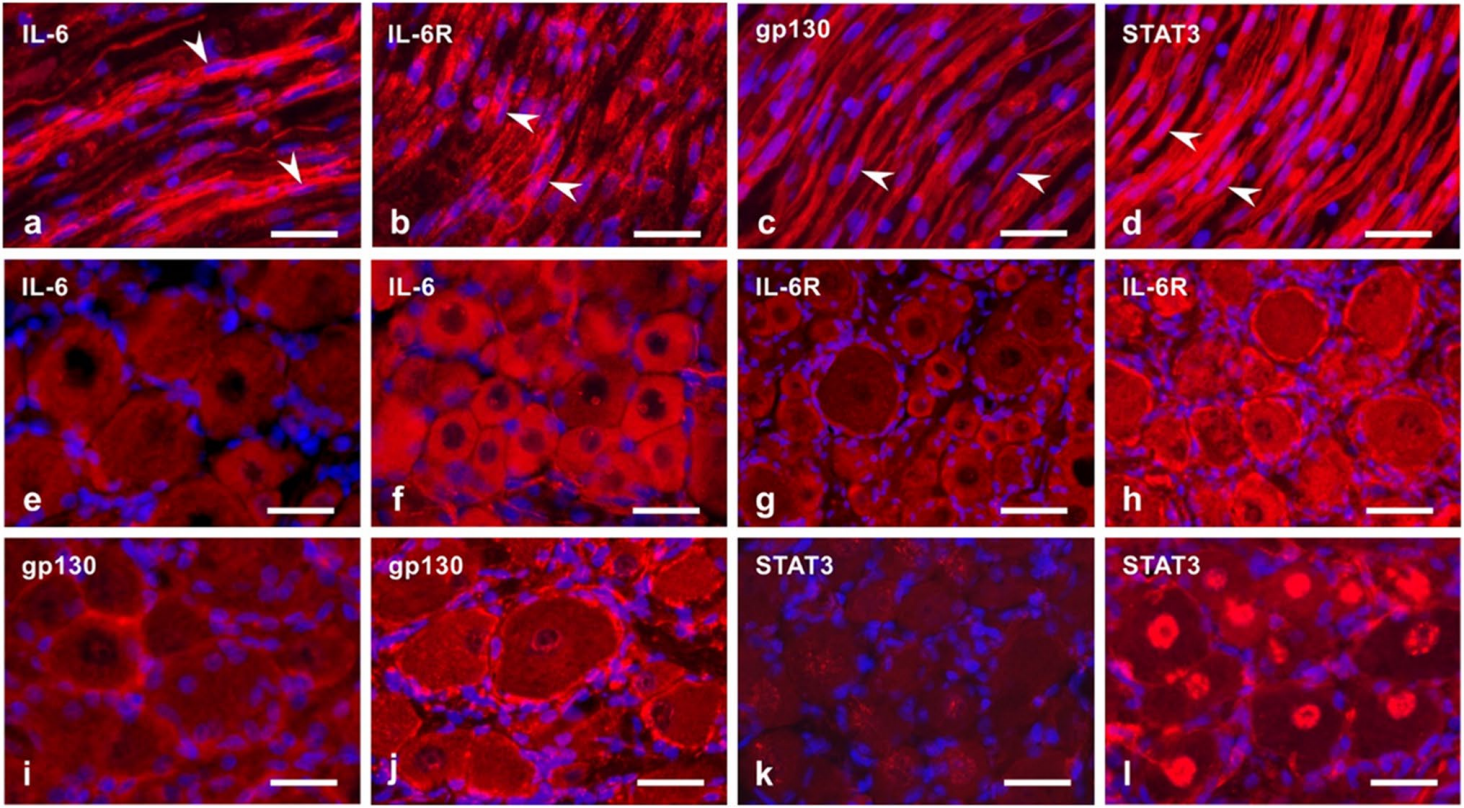
Longitudinal cryostat sections through rat sciatic nerve distal to compression for 14 days (**a**–**d**). The sections were immunostained with rabbit polyclonal anti-IL-6 (**a**), anti-IL-6R (**b**), anti-gp130 (**c**) or anti-STAT3 (**d**) (all red signals). Arrowheads indicate the position of activated Schwann cells that displayed immunopositive staining. Representative sections through the fourth lumbar dorsal root ganglion were removed from intact rats (**e**, **g**, **i**, **k**) and those operated on triplet ligature to compress the sciatic nerve for 14 days (**f**, **h**, **j**, **l**). The sections were immunostained with rabbit polyclonal antibodies against IL-6 (**e**, **f**), IL-6R (**g**, **h**), gp130 (**i**, **j**) or STAT3 (**k**, **l**). Cell nuclei were stained using Hoechst [scale bars, 35 µm (**a**–**d**) and 30 µm (**e**–**l**), respectively]

Additionally, immune mediators’ functions can also be demonstrated by the contribution of IL-6 and activation of STAT-3 signalling in the regeneration of primary sensory neurons (Dubový et al. 2019) (Fig. 2). Further, IL-6 also promotes regeneration of injured peripheral nerves (Hirota et al. 1996; Cámara-Lemarroy et al. 2010). However, it can also have deleterious effects if overproduced, as shown in sciatic nerve regeneration (Ma et al. 2019). Under these circumstances, it can also be accompanied by neuropathic pain (Zhou et al. 2016). Mechanistically, the intensity of hyperalgesia correlates here with the increased production of inflammatory mediators, including IL-6 (Patil and Testarelli 2021). This production can be reduced, for example, by cannabinoids and/or flavonoids, with a therapeutically relevant effect, i.e. reduced pain intensity in patients with hyperalgesia (Henshaw et al. 2021; Rao et al. 2021).

Incomplete or incorrect peripheral regeneration of sensory fibres is also associated with neuropathic pain induction (Cobianchi et al. 2014; Xie et al. 2017). From this point of view, immunomodulation (following nerve injury) to improve peripheral nerve regeneration efficiency remains one of the greatest challenges in regenerative medicine. It also has therapeutic potential due to its ability to relieve pain (Li et al. 2022).

## Autoimmunity – an injury due to auto-aggression

Autoimmune disease occurs when a specific adaptive immune response targets self-antigens, resulting in chronic inflammation-mediated tissue injury. Paul Ehrlich, a great German immunologist, famously coined the term horror autotoxicus for immune-mediated self-destruction (Ehrlich 1900). The immune system uses a plethora of mechanisms to damage tissues and organs in autoimmune diseases. However, the resulting tissue damage associated with fibrosis and impaired function is a surprisingly frequent feature of these deleterious scenarios.

### Scleroderma

Scleroderma is a well-known, potentially lethal devastating autoimmune disease of still not completely understood pathogenesis. It may also be called systemic sclerosis (SSc) because it is characterized by fibrotic changes not only in the skin, but also within visceral organs (van Caam et al. 2018). A substantial increase in the mortality rate of patients with SSc is related to cardiac disease, pulmonary fibrosis and pulmonary hypertension. As this maladaptive immune response develops, it is usually impossible for the immune effector cells to entirely eliminate the antigen. Currently used immunosuppressive treatments are only partially effective in the prevention of disease progression. This treatment is inefficient in the removal of already present fibrous tissue.

Histological examination of skin biopsies of patients with SSc revealed changes in the collagen fibre bundles and increased presence of SMA-positive cells in the dermis. The fibrosis in scleroderma is ultimately driven by myofibroblasts (van Praet et al. 2011). These myofibroblasts are particularly resistant to apoptosis. Their number in the dermis positively correlates with abundant collagen overproduction, enhanced ECM stiffness and up-regulated pro-fibrotic cytokines, resulting in elevated skin hardness and disease progression (Ferreli et al. 2017). In the background of tissue inflammation, myofibroblasts are autonomously activated with TGF-β as the critical factor responsible for SMAD-dependent signalling (Ramirez et al. 2006). However, it has also been noted that the TGF-β signalling loop is insufficient to explain the persisting myofibroblast phenotype (Leask 2013).

IL-6 acts as a pro-fibrotic factor in scleroderma (Pedroza et al. 2011). It is increased in patients with scleroderma and significantly correlates with the disease severity scoring system (Shima et al. 2010). Substantial evidence has been gathered to support the role of IL-6 in the disease activity and the development of cardiopulmonary manifestations in patients with SSc (Distler et al. 2020). In this context, serum IL-6 levels have appeared to be predictive of early disease progression in patients with interstitial lung disease.

It was suggested recently that TGF-β and IL-6 pathways could cooperate in fibrous disorders, e.g. idiopathic pulmonary fibrosis. There is a growing body of evidence that lung fibroblasts express high baseline levels of both canonical and IL-6 trans-signalling components, leading to indirect TGF-β pathway activation and disease progression (Epstein Shochet et al. 2020). Therefore, IL-6 inhibitors may prevent early lung disease (Khanna et al. 2020). Although tocilizumab in a phase III clinical trial did not achieve the reversal endpoint of the skin fibrosis (assessed by modified Rodnan skin score), results for the secondary endpoint (predicted forced vital capacity) indicated that the treatment might preserve the lung function in persons with early involvement.

In addition, it also seems likely that combining different immunotherapies in treating scleroderma may be beneficial for patients. In a murine model, combined CD47 and IL-6 blockade reversed skin fibrosis and led to rapid elimination of ectopically transplanted scleroderma cells (Lerbs et al. 2020).

### Rheumatoid arthritis

Rheumatoid arthritis, an autoimmune inflammatory disease, is a long-term condition that is characterized by pain, swelling and stiffness of the affected joints. In addition to immune cells, activated mesenchymal cells actively contribute to pathological tissue repair of joints, leading to pannus formation by perpetuation of an autoimmune reaction (Schuster et al. 2021). In rheumatoid arthritis, fibroblast-like synoviocytes are the most common cell type at the pannus–cartilage junction. Similarly to activated fibroblasts/myofibroblasts, these synoviocytes in rheumatoid arthritis contribute to the joint destruction via production of cytokines, chemokines and matrix-degrading agents (Bustamante et al. 2017). Like other autoimmune diseases, rheumatoid arthritis is frequently accompanied by extra-articular manifestations, namely lung fibrosis, with numerous myofibroblasts present in fibrotic tissue (Popper et al. 2021).

Rheumatoid arthritis is frequently associated with the elevation of IL-6. Pleiotropic effects of IL-6 participate in the control of articular and extra-articular pathologies (Jarlborg and Gabay 2022). Although IL-6 is up-regulated and may result in the cytokine storm, it is relatively rare in these patients (Mehta et al. 2020). On the other hand, the progression of the autoimmune disease resulting in cachexia and severe psychiatric issues (depression and anxiety) is common. It was suggested that it is mechanistically due to the up-regulation of bioactive factors such as TNF-α, IL-1, IL-6, IL-17 and C-reactive protein (CRP) (Fakra and Marotte 2021; Chimenti et al. 2021; Ollewagen et al. 2021). This concept has been confirmed, as anti-IL-6 therapy (tocilizumab or sarilumab) exhibited good efficacy and tolerability in rheumatoid arthritis with poor response to conventional treatment options (Yip and Yim 2021). Importantly, administration of these drugs treats the affected joints, but also alleviates extra-articular manifestations occurring in the patients. Consequently, stabilization of lung fibrosis and reduction of cachexia were also reported (d’Alessandro et al. 2020; Patsalos et al. 2020).

## Cancer

Tumours mimic the complex structure of physiological organs. Tumours are structured and tightly orchestrated ecosystems formed jointly by malignant and non-malignant cells (Lacina et al. 2018). The elements of the tumour stroma [cancer-associated fibroblasts (CAFs), endothelial and immune cells and ECM] are not only bystanders, but are active participants of oncogenic processes. Cells within the cancer ecosystem also communicate directly via intercellular contacts or indirectly by releasing paracrine products. In many aspects, the tumour stroma resembles granulation tissue. Indeed, the tissue microenvironment of wounds (Čoma et al. 2021) and autoimmune disorders share many general features and regulatory mechanisms with the tumour stroma. Furthermore, the dynamic cellular interplay involving numerous cell types of the immune system in inflammation can also be relevant in cancer. Not surprisingly, an almost identical set of bioactive molecules can be observed during wound healing and cancer growth/spreading.

Precise targeting of tumour cells has been a primary goal of oncological therapy for decades. More recently, the development of immune checkpoint inhibitors, a novel class of immunotherapy drugs, has heralded a new era in cancer therapy (Smetana et al. 2020a; Genova et al. 2022; Yi et al. 2022). This therapeutic targeting of the tumour-resident immune cells proved to be a remarkably successful tool to treat cancer. In 2018, the Nobel Prize was awarded to Tasuku Honjo and James Allison for their contribution to this strategy.

On the other hand, cancer-associated fibroblasts (CAFs), another essential component of the tissue microenvironment (TME), have been largely overlooked in current treatment protocols. CAFs represent a fundamental bioactive part in many tumours. CAFs stimulate migration and poor differentiation of cancer cells (Kolář et al. 2012; Trylcova et al. 2015; Jobe et al. 2016, 2018). On the other hand, CAFs may also suppress the tumour microenvironment by lowering hepatocyte growth factor production, resulting in reduced tumour size and formation of metastasis (Pallangyo et al. 2015). Although CAFs influence the biological properties of tumours and support the process of metastasis formation, specific targeting of CAFs has not yet been widely employed in clinics.

In many aspects, CAFs share multiple features of myofibroblasts and represent one of the most prominent non-malignant cell types of many tumours producing bioactive factors (Plzák et al. 2019). CAFs may arise from normal resident tissue fibroblasts (recruited and activated by malignant cells), similarly to myofibroblasts arising from normal dermal fibroblasts in a wound. However, CAFs can also be derived from a wider panel of potential precursors such as mesenchymal stem cells, adipocytes, stellate cells, circulating fibrocytes, pericytes, mesothelial cells and epithelial and endothelial cells following exposure to potent pro-inflammatory factors (Liu et al. 2019), including but not limited to IL-1β, TGF-β and platelet-derived growth factor (PDGF) (Vokurka et al. 2022).

**Fig. 3:**
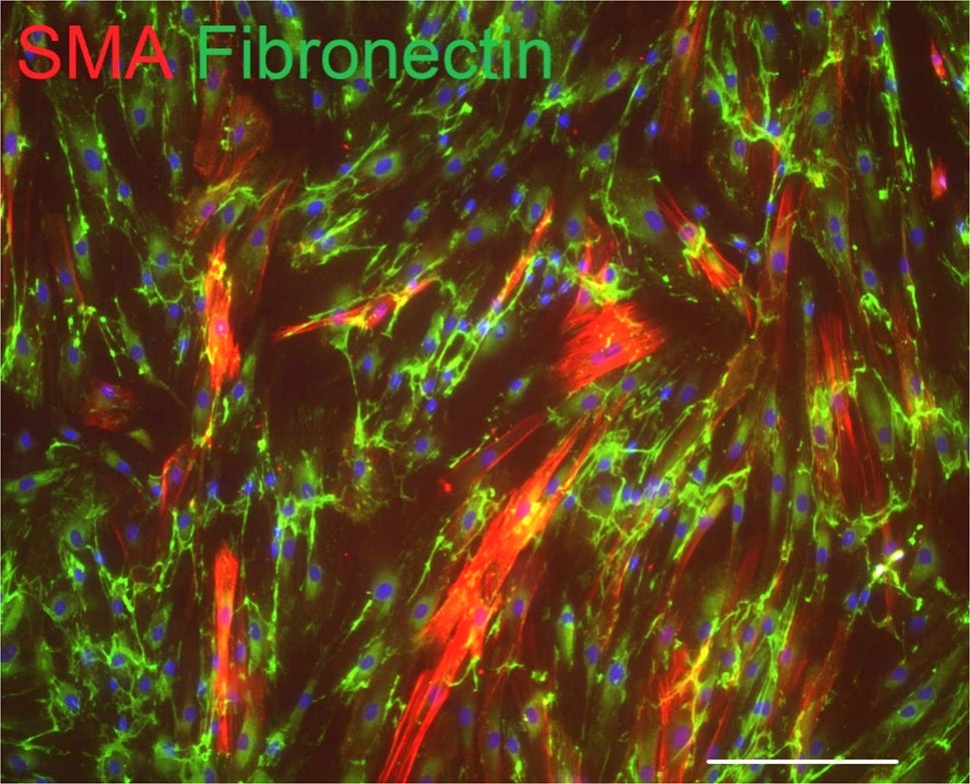
Numerous fibroblasts isolated from the skin metastasis of cutaneous malignant melanoma exhibit α-smooth muscle actin (SMA, red signal). These fibroblasts/myofibroblasts also produce extracellular matrix molecule fibronectin (green signal). Nuclei were counterstained with DAPI (scale bar, 100 µm)

So far, there is no specific marker to identify all CAF subpopulations available for research and/or diagnostic purposes. The effects seem to be mediated especially by IL-6, IL-8, CXCL-1, CXCL-8 and specific ECM components. Mounting evidence illustrates that CAFs are a very heterogeneous population of cells (Kalluri 2016). The expression of fibroblast markers is extremely heterogeneous and varies strongly between different CAF subpopulations. Therefore, for reliable definition and detection of CAFs, it is recommended to use a broad panel of markers including produced cytokines and ECM proteins. Furthermore, this panel should also consider the origin of fibroblasts and the type of cancer. SMA (Fig. 3) and fibroblast activation protein alpha (FAP) seem to be widely accepted and stable markers of CAFs, separating CAFs from the larger pool of fibroblasts present in the body (Nurmik et al. 2020; Vokurka et al. 2022).

In this context, tumours with unfavourable prognosis (e.g. metastatic seminoma) have demonstrated a notable increase in pro-inflammatory activity, including IL-6 signalling (Nestler et al. 2022). Moreover, the production of pro-inflammatory mediators by CAFs can be significantly stimulated by exosomes released by cancer cells (Strnadová et al. 2022), indicating synergistic effects. However, whether these mechanisms are suitable as targets for novel therapies has to be answered in further studies in the future.

Mediators of intercellular crosstalk of the cancer ecosystem can also enter blood vessels and reach distant tissues with the ability to modulate their functions. It has been shown that IL-6 and CXCL-8 produced by CAFs can thus be detected in the circulating fluids (e.g. serum), making them useful for diagnostic/prognostic purposes to monitor disease progression. This was confirmed on a clinical basis in patients suffering from cutaneous malignant melanoma as well as ovarian, squamous cell, breast and colorectal cancers (Xu et al. 2016; Kučera et al. 2019; Rezaei et al. 2019; Wang et al. 2021; Amer et al. 2021; Paccagnella et al. 2022). For example, in patients with breast cancer, increased levels of IL-4, IL-6, IL-8, IL-10, CCL-2 and IFN-γ are associated with lower survival rates (Paccagnella et al. 2022).

In line with this evidence, IL-6 and other inflammation-supporting factors have an essential role in generating a so-called premetastatic niche. These tissue domains offer a permissive microenvironment allowing circulating tumour cells to harbour and foster in the sensitive initial period before they gain full control over the new metastatic site (Kodet et al. 2020; Li et al. 2020). Secretion of IL-6 and premetastatic niche formation is controlled by exosomal miR-21. It was shown in an animal model that miR-21 silencing reduces the level of IL-6 and minimizes metastasis of colorectal cancer to the liver (Shao et al. 2018).

The terminal stage of malignant disease is frequently associated with cancer-related cachexia. It is explained as a systemic effect of pro-inflammatory factors such as IL-6, CXCL-8 and TNF-α in skeletal muscle, adipocytes and hepatocytes (Kasprzak 2021; Paval et al. 2022). In many aspects, cancer-associated cachexia seems to be similar to that of a cytokine storm described in several viral infections (including COVID-19). High levels of IL-6 and other cytokines lead to deterioration of patients’ organs and cause exhaustion (White 2017; Yehia et al. 2021). Interestingly, moderate physical exercise can prevent, at least partially, the cancer-induced trend of cachexia (Wood et al. 2022). The increased level of IL-6 also influences the hypothalamus–pituitary–adrenal axis linking cancer with depression (Jehn et al. 2010) and reduction of food intake (anorexia) (Molfino et al. 2017). We may conclude that enhanced levels of pro-inflammation factors are associated with more severe psychiatric symptoms and further deteriorate the emotional wellbeing of patients with cancer (Santoft et al. 2020).

## COVID-19

COVID-19 is a pandemic respiratory infection caused by the SARS-CoV-2 coronavirus. It is frequently accompanied by severe bilateral pneumonia resulting in acute respiratory distress syndrome. It can be fatal for many patients, especially those suffering from comorbidities associated with advanced age. Lung damage and respiratory problems can persist even after recovering from the acute disease (also known as long COVID-19). In this context, cases of lung transplantation for respiratory failure due to COVID-19 were also reported (Aul et al. 2021; Hall et al. 2021).

Lung autopsy of individuals who died from COVID-19 revealed three main histopathological patterns (Valdebenito et al. 2021):

- (A) Haemorrhage with minimal immune infiltration and extensive thrombus,
- (B) Heavy immune infiltration without thrombus formation, and
- (C) Combination of type 1 and 2 with proliferation of fibroblasts and development of fibrosis.

Pulmonary fibrosis is an immensely interesting and potentially fatal feature resulting from this disease. This fibrosis causes gas-exchange impairment, profoundly affecting the quality of some surviving patients’ lives. However, the pathophysiological background of the resulting fibrosis remains relatively poorly understood. We believe that this is a critical aspect, and it is worthy of closer analysis.

Mesenchymal stem cells (MSCs) are present in nearly all tissues/organs of the body. MSCs are multipotent stem cells with self-renewal capacity. MSCs can generate clonal populations with the ability of multilineage differentiation. Thus, MSCs play an essential role in the repair and generation of tissues (Kavianpour et al. 2020). Notably, MSCs possess broad immunoregulatory properties. This is possible via interactions with the innate and adaptive immune systems. MSCs can cause immune suppression of many processes. Indeed, MSCs can attenuate the cytokine storm in severe/critical COVID-19, as shown in several studies (Kaffash Farkhad et al. 2021).

**Fig. 4:**
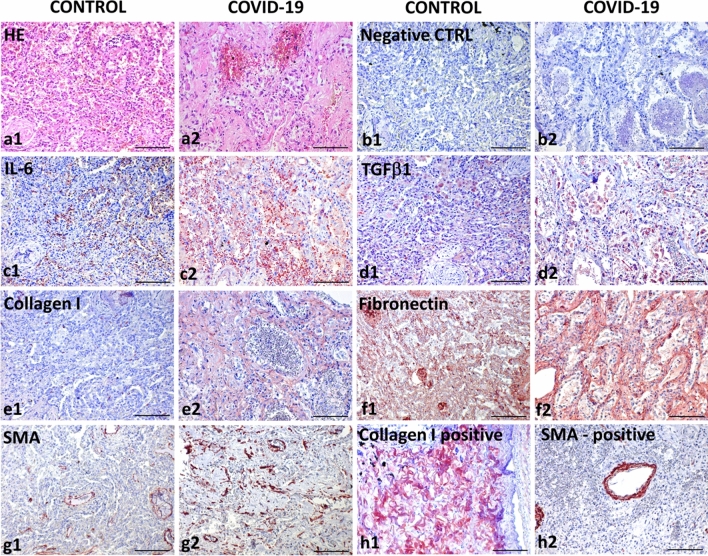
Comparative analysis of a lung abscess (**a1**–**g1**) and lungs destructed by COVID-19 (**a2**–**g2**). The structure of the lungs is seriously altered in patients suffering from both diseases, as visible in the figures depicting haematoxylin and eosin staining (a1, a2). Negative control using isotype control antibodies (**b1**, **b2**) is included to confirm specificity of the following immunohistochemical reactions using horse radish peroxidase (HRP)-tagged antibodies and AEC (red) substrate. Expression of IL-6 was significantly lower in the lungs of the patient without COVID-19 (**c1**) than in the lungs of the patient with COVID-19 (**c1**). A similar trend was observed for the expression of TGF-β1 (**d1**, **d2**), type I collagen (**e1**, **e2**) and fibronectin (**f1**, **f2**). While in the non-COVID lungs SMA expression was limited to the smooth muscle cells in the wall of vessels and bronchiole (**g1**), numerous SMA-positive myofibroblasts were found in the COVID-19 lungs (**g2**). The presence of type I collagen in peribronchial fibrous tissue (**h1**) and SMA in vessels (**h2**) was visualized in the positive control confirming reactivity of primary antibodies. Nuclei were counterstained with Gill’s haematoxylin (scale bar, 100 µm)

However, mammalian lungs, including human, contain heterogeneous subpopulations of mesenchymal cells that can modulate pulmonary fibrosis and functions (Liu et al. 2021). One of these populations are lung lipofibroblasts, cells with a remarkable ability to store lipid droplets. In collaboration with myofibroblasts, lipofibroblasts also participate in alveolar regeneration (Ushakumary et al. 2021). Under specific conditions such as hypoxia, lipofibroblasts can be extensively transformed into myofibroblasts and participate in the pathogenesis of lung fibrosis (Rehan and Torday 2003, 2014; Kruglikov and Scherer 2020). This process seems to be regulated by IL-6 and TGF-β (Fig. 4). Of note, it was reported that metformin alters the transition of lipofibroblasts into myofibroblasts. There is also evidence that this widely used oral antidiabetic drug minimizes lung fibrosis progression (Kheirollahi et al. 2019). Following viral injury of the lung alveoli and blood vessels, TGF-β and IL-6 initiate transformation of resident fibroblasts into myofibroblasts (Fig. 4). Moreover, these pro-inflammatory cytokines also induce EMT, EndMT and generation of myofibroblasts (Giacomelli et al. 2021). Of note, circulating fibrocytes (bone marrow-derived cells) were also suggested as a population potentially participating in myofibroblast induction (Ghanem et al. 2021).

The role of mesenchymal cells in fatal COVID-associated pneumonia is supported by recent data obtained using single-cell sequencing techniques. Analysis of tissue samples from affected lungs demonstrated, as expected, a broad spectrum of significantly activated infiltrating immune cells (such as macrophages). However, microscopic analysis also revealed a surprisingly large proportion of fibroblasts and myofibroblasts (Delorey et al. 2021; Wendisch et al. 2021). These studies also highlighted a remarkably limited ability of lung alveoli to self-renew following viral infection, such as infrequent local hyperplasia of alveolar type II cells.

It is known that SARS-CoV-2 enters the cells via angiotensin-converting enzyme 2 (ACE2) (the spike protein of the virus binds to ACE2) on the cell surface. ACE2 is expressed mainly on alveolar type II cells, making the lungs the primary target for the infection (Zou et al. 2020). ACE2 and transmembrane serine protease 2 (TMPRSS2) mediate virus internalization. These molecules are co-expressed in type II pneumocytes, ileal absorptive enterocytes and nasal goblet secretory cells (Ziegler et al. 2020). SARS-CoV-2 prefers activation by TMPRSS2, but if the target cells express low levels of TMPRSS2 or if the virus–ACE2 complex does not encounter TMPRSS2, the complex can be internalized through clathrin-mediated endocytosis (Karthika et al. 2021). Although type I alveolar cells are not primarily targeted by SARS-CoV-2, they develop from the type II cells. Thus, the type I pneumocyte population can be depleted after type II cells are destroyed owing to the viral cytopathic effect. Type II cells infected by SARS-CoV-2 produce high levels of IL-6 (Gajewski et al. 2021). The massive infection and subsequent inflammation-induced swelling also increase mechanic tension that stimulates production of TGF-β by type II pneumocytes. The increased mechanical stress facilitates the transition of fibroblasts/lipofibroblasts into myofibroblasts. This process is of critical importance for respiratory failure and development of pulmonary fibrosis (Wu et al. 2020). In combination with an increase in the fibroblast number and consequent ECM deposition (rich in fibronectin and collagen), the risk of developing severe/critical lung injury resulting in fibrosis and even death increases (Fig. 4) (Bridges et al. 2022).

It has been well demonstrated that the hyperactivation of the immune system accompanied by the enormous increase in the production of bioactive factors such as a panel of cytokines including IL-6, chemokines and growth factors leads to cytokine storm and is closely related to the severity of COVID-19 disease (Pedersen and Ho 2020; Smetana and Brábek 2020). Although understanding the pathogenesis of cytokine storm allows us to unravel risk factors for the condition and substantiates novel therapeutic strategies, evidence disputing the entire concept of cytokine storm has also been presented. Indeed, exceptions to this scenario are possible, as immunological responses strictly reflect the setting of an individual immune system. Indeed, some patients with severe COVID-19 exhibited no significant elevation of IL-6 (Wu et al. 2021). However, there is robust evidence that the severity of the COVID-19 disease – namely the level of lung damage – can be monitored via the detection of inflammatory markers in the serum, i.e. IL-6, CRP, procalcitonin and TNF-α represent the most reliable markers of the disease progression (Parimoo et al. 2021; Mardani et al. 2022). IL-6 has broad systemic effects and alters the metabolism, including age/cancer-related muscle wasting and cachexia (Pettersen et al. 2017).

Last but not least, research has also pointed to sex differences in the course of COVID-19 infection, since most countries showed disproportionately higher deaths and higher case fatality rates among men (Dehingia and Raj 2021). Sex differences refer to the biological attributes, including hormonal, immune and inflammatory responses to infection, that potentially influence the severity and outcomes of the infection (Ya’qoub et al. 2021). Oestrogens promote both innate and adaptive immune responses, potentially leading to faster clearance of pathogens, less severe symptoms in women and a more robust immune response to vaccines (Takahashi and Iwasaki 2021). Moreover, oestrogens reduce the risk of immune system hyperactivation during cytokine storms and the expression of ACE2 necessary for SARS-CoV-2 internalization. Therefore, oestrogens can protect women against serious disease progression (leading to lower mortality of women) (Abramenko et al. 2021).

## Role of IL-6 and other inflammation-supporting factors

Our previous papers deal in detail with the mechanisms and cellular sources of the pleiotropic cytokine IL-6 (Lacina et al. 2019, 2021; Brábek et al. 2020; Čoma et al. 2021; Španko et al. 2021). Almost all cells in the human body can produce IL-6. The signalling is initiated via a heterodimeric complex consisting of IL-6 α-receptor (IL-6R) and signal-transducing β-subunit glycoprotein 130 (gp130) (Wolf et al. 2014). IL-6R may be found in soluble and membrane-bound forms, which allows distinction between IL-6 classic signalling (via the membrane-anchored IL-6R) and IL-6 trans-signalling via the soluble IL-6R (sIL-6R) (Rose-John 2012). Evidence suggests that IL-6 signalling via sIL-6R induces pro-inflammatory activity. In contrast, the anti-inflammatory activity of IL-6 is mediated via classic signalling. Therefore, specific inhibition of the sIL-6R pathway presents a valuable option for treating inflammatory diseases (Jones et al. 2011).

For example, a lack of IL-6 (and tumour necrosis factor receptor-1) results in more severe impairment of the innate immune response. IL-6 also stimulates proliferation of many cells and thus is essential for proper wound healing (Albrecht et al. 2016). An imbalance of pro-/anti-inflammatory cytokines may interfere with normal healing, whereas sustained elevated levels of IL-6 may lead to various complications (Rosa et al. 2008). Similarly, high levels of IL-6 are typical of autoimmune diseases such as rheumatoid arthritis or SSc. The cytokine also stimulates proliferation as well as migration/spreading of malignant cells, and induces low differentiation, which is an important hallmark of cancer resistance to conventional therapy (Jobe et al. 2016, 2018).

Why has IL-6 attracted so much attention in recent years? As we face population ageing in Western countries, the incidence of malignant and rheumatic diseases increases (Smetana et al. 2016; Iorio et al. 2021). Ageing also affects the immune system, referred to as immunosenescence. The altered setting of human immune response in the elderly is associated with systemic low-grade chronic inflammation, a phenomenon termed inflammaging (Franceschi et al. 2018; Brábek et al. 2020). It coincides with the accumulation of acquired mutations, partially due to the reduced gene repair capacity (Smetana et al. 2016) or long-term exposure to oxidative stress present in our environment (Bauer and de la Fuente 2016). According to a broadly accepted concept, inflammaging represents age-dependent deregulation of immune functions. It reduces adaptive immunity and is characterized by reduction of the immune response. It is also accompanied by up-regulation of pro-inflammatory cytokines, especially IL-6 and TNF-α. This mechanism may be related to numerous age-related health issues, including susceptibility to develop a more severe course of COVID-19 (Barbé-Tuana et al. 2020; Santoro et al. 2021). It has been well documented that individuals over 60–65 years of age are at a higher risk of COVID-19-related mortality. The concept of inflammaging is well supported by the immune response in survivors of childhood cancer who had received chemotherapy and/or radiation therapy as a consequence of premature ageing (Rossi et al. 2021). Children accumulate senescent cells, exhibit numerous DNA mutations and extensively produce reactive oxygen species that do not match their age. This may result in premature failure of vital organs (Rossi et al. 2021). Also typical of inflammaging is reshaping of the cytokine expression profile, with progressive tendency towards an inflammation-supporting phenotype (up-regulation of CRP, IFN-γ, IL-1, IL-6, IL-8, IL-12, TNF-α) (Rea et al. 2018).

Among these factors, IL-1, IL-6 and TNF-α remarkably support cancer growth and spreading (Jobe et al. 2016; Bleve et al. 2022; Strnadová et al. 2022). IL-6 represents a direct link between autoimmune disorders, cancer and age-related diseases, thus offering an attractive therapeutic target (Iorio et al. 2021). From this perspective, repurposing already available drugs for other clinical applications seems to be a highly rational approach. Therapeutic blocking of IL-6 signalling available for arthritis treatment can thus be an inspiring and readily achievable strategy for cancer therapy as well (Španko et al. 2021). However, from recently limited clinical experience, it seems that blocking of IL-6 is not sufficient to treat tumours. However, on the basis of experimental data, we can hypothesise that simultaneous blocking of other factors might be more promising, e.g. simultaneous inhibition of IL-6 and IL-8 (Jobe et al. 2016; Plzák et al. 2019; Zhang et al. 2022). For example, the combination of bazedoxifene (gp130 inhibitor) and paclitaxel seems to be potentially suitable for the therapy of ovarian cancer (Park et al. 2022).

It has been shown that the process of inflammaging can be promoted by external factors. For instance, chronic infection induced by cytomegalovirus (CMV) acts in a synergic manner, accelerating the natural process of inflammaging (Bauer and de la Fuente 2016). CMV infection together with immunosenescence and inflammaging contributes to hypersecretion of inflammation-supporting cytokines, resulting in a hyperinflammatory syndrome/cytokine storm with a severe-to-fatal course of COVID-19 in the elderly (Müller and di Benedetto 2021). The mediators of inflammation, such as IL-6 and TNF-α, also activate blood platelets, resulting in abnormal blood clotting – another severe complication of COVID-19 (Xu et al. 2021). To conclude this section, IL-6 has a central regulatory role in numerous physiological and pathological processes and thus represents an immensely promising target for therapeutic interventions.

## The therapeutic consequences of COVID-19

**Fig. 5:**
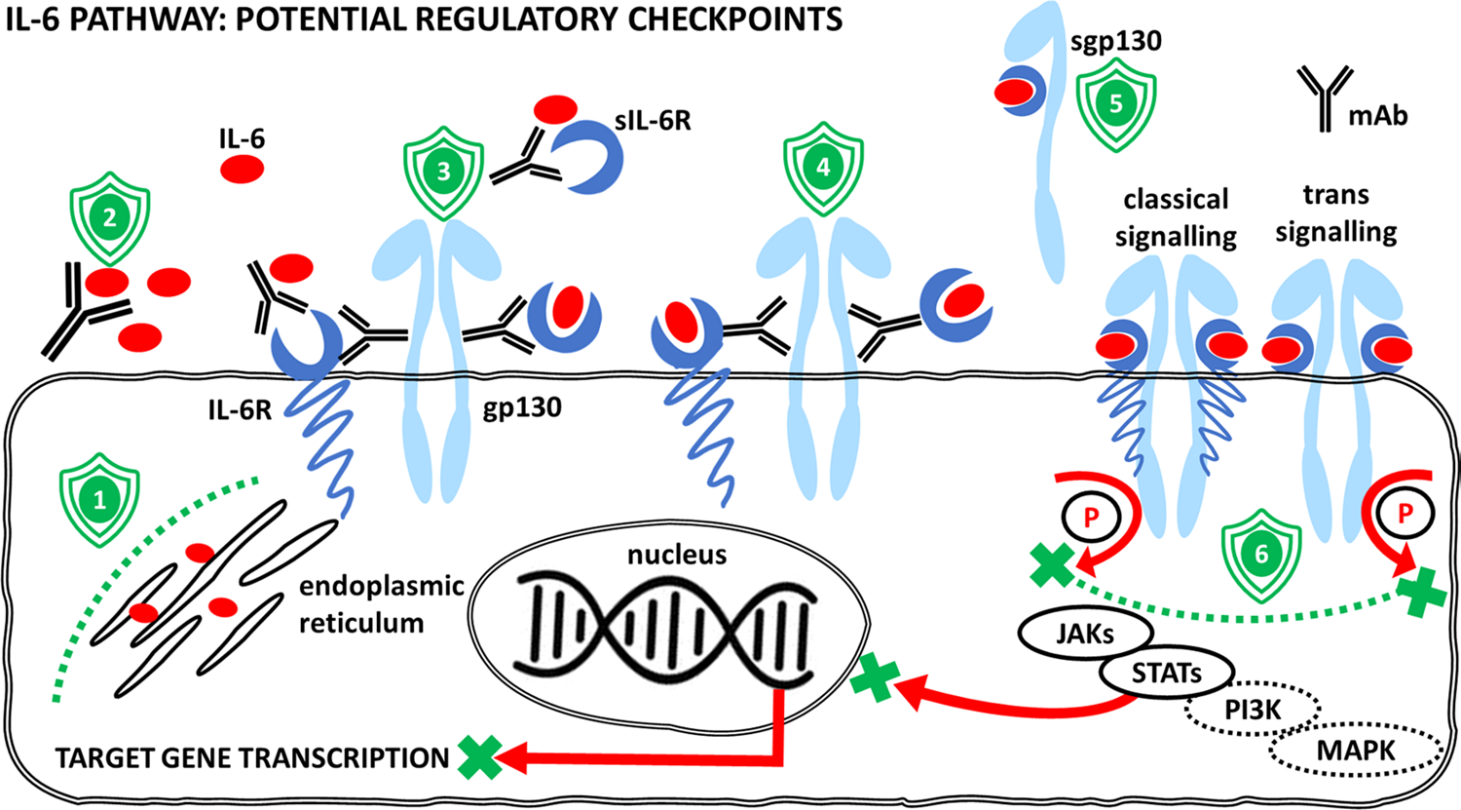
Interleukin-6 (IL-6) signalling can be therapeutically regulated at several checkpoints. (1) The production of IL-6 can be diminished (e.g. using curcumin). (2) Once released, the bioavailability of IL-6 can be diminished by neutralizing antibodies (mAb) (e.g. siltuximab). (3) Another therapeutic approach can be based on targeting the interleukin-6 receptor (IL-6R) and/or its soluble form (sIL-6R) (e.g. tocilizumab, sarilumab). This can prevent IL-6R from binding to the cytokine IL-6 and formation of the active cytokine–receptor complex. Alternatively, antibodies or small-molecule inhibitors against the receptor or signal transducer gp130 can prevent the binding of the activated complex (IL-6/IL-6R or IL-6/sIL-6R) to signal transducer glycoprotein gp130 (e.g. bazedoxifene). (4) Antibodies raised against epitopes of signal transducer gp130 can also prevent the binding of the activated complexes (IL-6/IL-6R or IL-6/sIL-6R) (e.g. B-R3). (5) The soluble form of the signal transducer molecule (sgp130) binds the active complex of IL-6/sIL-6R (e.g. olamkicept). This has an inhibitory effect on trans-signalling by reduction of available sIL-6R. It can also help to sequestrate free IL-6. (6) Inhibitors of gp130 kinase activity prevent phosphorylation (P) of downstream signalling molecules (JAKs, STATs, PI3K, MAPK) (e.g. baricitinib), with their consequent translocation to the nucleus, where they regulate target gene transcription. Examples of drugs were selected from Španko et al. (2021)

Targeting the IL-6 signalling represents a plausible therapeutic strategy for several autoimmune diseases and cancers (Brábek et al. 2020; Španko et al. 2021) (Fig. 5). In recent years affected by the COVID-19 pandemic, the number of potential indications of this strategy has increased. The European Medicines Agency recently approved anti-IL-6 receptor monoclonal antibody tocilizumab to treat patients with severe and critical forms of COVID-19. The drug is recommended for patients with COVID-19 who progress to respiratory failure within 72 h after admission to hospital despite corticosteroid treatment. The guidelines for COVID-19 therapy also include sarilumab, which can be used instead of tocilizumab in case of its shortage.

Preliminary data from a single-centre study on 22 patients with COVID-19 performed at the Department of Infectious Diseases, First Faculty of Medicine, Military University Hospital Prague and Charles University, confirmed the positive effect observed in both tocilizumab-treated (*n* = 18) and sarilumab-treated (*n* = 4) groups of severe and critical forms of COVID-19. Only two fatal cases were recorded among the included sample of patients (*n* = 22), indicating in-hospital mortality slightly below 10%, which is lower than that reported in the first and second waves of COVID-19 (Gray et al. 2021; Martinez-Guerra et al. 2022) and correlates well with published data (Gupta et al. 2022).

The approval of EMA has opened a new horizon for anti-COVID-19 therapy. Perhaps it will also be soon translated to innovative therapeutic approaches to other viral infections accompanied by a cytokine storm. Potential therapeutic strategies (including low-molecular-weight inhibitors) can regulate cytokine production, block the active site of IL-6 binding, inhibit IL-6 receptor complex activity and/or block intracellular signalling (Kang et al. 2019). These drugs were mainly designed to treat autoimmune diseases (e.g. rheumatoid arthritis) or proposed for experimental cancer therapy; some drugs are already under investigation in several clinical trials in head and neck cancer, as reviewed in our recent article (Španko et al. 2021). Therefore, their use as new anti-COVID-19 therapeutics can be expected, but immediate clinical application warrants further careful evaluation.

As presented previously (Abramenko et al. 2021), natural oestrogens and some other oestrogen receptor modulators have improved the outcome of severe COVID-19, also inhibiting the interaction of IL-6 with its receptor. This mechanism reduces the risk of developing the cytokine storm. Some of these molecules, such as bazedoxifene or raloxifene, recognize the IL-6 receptor complex by interaction with gp130 and thus reduce the ability to bind IL-6. Both agents also recognize the viral spike protein and minimize virus entry to the target cells or interact with the viral main or papain-like protease inhibiting virus proliferation (Song et al. 2020; Brábek et al. 2020; Abramenko et al. 2021). This makes these drugs excellent candidates for repurposing. Numerous reports have indicated several candidate drugs (even broadly available) for COVID-19 treatment based on the inhibition of sIL-6 signalling. For example, anti-cholesterol medication lovastatin can reduce the level of IL-6. Indeed, it was reported that the COVID-19-related mortality of patients on statin treatment was significantly reduced (Karampoor et al. 2021).

## Conclusion

The wound healing of various tissues is a precisely controlled process regulated by evolutionarily conserved signalling pathways. In the present review, we compared the general aspects of inflammatory/immune responses and regulation of this process in wounds, tumours, selected autoimmunity disorders and COVID-19 (Table 1).

**Table 1:** Comparison of wound healing, cancer and COVID-19

Parameter	Wound healing	Rheumatoid arthritis	Cancer	COVID-19
Recruitment of immune cells	Yes	Yes	Yes	Yes
Effect of immune cells on non-immune cell proliferation	Yes (fibroblasts + re-epithelization)	Yes (fibroblasts)	Yes (cancer cells + their migration)	Yes (fibroblasts)
Cytokine storm	Almost not	Yes (rare)	Yes (cancer cachexia)	Yes
IL-6 elevation	Yes	Yes	Yes	Yes
Stromal reaction	Yes (granulation tissue)	Yes (pannus)	Yes (cancer stroma)	Yes (inter-alveolar septa)
ECM production	Yes	Yes	Yes	Yes
Myofibroblasts	Yes	Yes	Yes	Yes
Final effect	Wound closure	Progression of articular/extra-articular signs	Growth + metastasizing	Disease progression + fatal termination

We aimed to underscore the conceptual hypothesis of transition of the chronic course of a pathological healing-like process into an inflammation-resolving status that is beneficial for the patient. Specific regulation of inflammation occurs via controlled release of particular sets of cytokines/chemokines and immune cell populations, i.e. with factors critically relevant to health maintenance. The damage observed in autoimmune diseases develops owing to the failure of regulation and tolerance, resulting in a sustained, long-lasting course of inflammation with detrimental structural and functional effects on the whole organism. However, an acute hyperactive destructive immune over-response (cytokine storm) also results in deleterious effects, e.g. on the lungs of patients suffering from severe/critical COVID-19 disease. Furthermore, deregulated production of immune factors was associated with terminal cachexia in many patients with cancer. The inflammatory mediators are produced not only by recruited leucocytes, but also by local mesenchymal cells (including fibroblasts). Activated fibroblasts proliferate, transit to myofibroblasts and produce a huge amount of bioactive molecules regulating the inflammatory response. Both chronic and acute inflammatory over-reactions may consequently induce fibroproliferative changes. Fibrosis leads to deterioration of organ structure and impairment of organ function, thus presenting a devastating clinical issue. The context defines the outcome; Matthew 7:16 “You Will Know Them by Their Fruits”. Together, the role of stromal fibroblasts in the context of microenvironment regulations deserves serious attention, and their potential to become the targets of novel therapeutic concepts should be considered in multiple diseases.

**Fig. 6:**
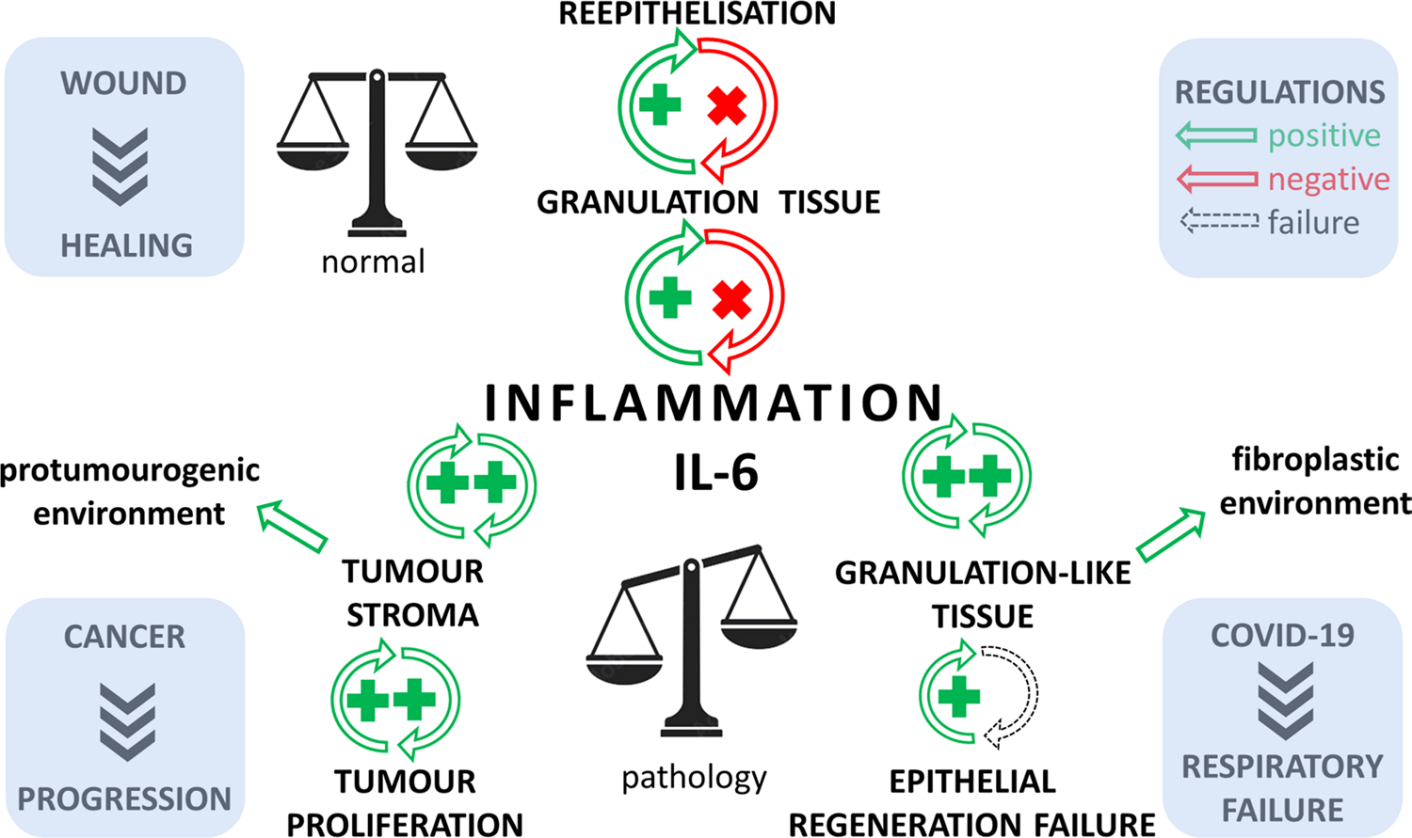
The diagram demonstrates the role of inflammation (with emphasis on IL-6) in wound healing, cancer and severe/critical COVID-19 infection. While wound re-epithelization arrests the activity of granulation tissue and reduces inflammation, this negative feedback fails to act in the case of cancer and severe/critical COVID-19, worsening the prognosis of patients

In summary, our review points out that organisms react in different pathological situations according to a uniform scenario using conservative pathways, as observed during wound healing, in selected autoimmune diseases, cancer and COVID-19. If poorly regulated, this can all be unfavourable and even fatal. All these situations are associated with inflammatory processes, including the involvement of both innate and adaptive immune responses. While re-epithelization of wounds negatively interferes with the activity/growth of the granulation tissue, in cancer, chronic inflammatory disorders or severe infections involving cytokine storm, a similar inhibitory feedback is not elicited (Fig. 6).

Therefore, detailed understanding of the mechanisms regulating wound healing and driving autoimmunity and molecular signalling pathways in the tumour ecosystem provides immensely valuable information for other clinically relevant applications as well. In particular, the COVID-19 pandemic allowed the development of a strategy to rapidly repurpose available drugs. Almost instantly, this offered various therapeutic options for the management of hyperactivation of the immune system (e.g. anti-IL-6 therapy). In line with this experience, similar approaches may also follow as novel therapies for other, seemingly unrelated diseases.
